# Effects of Chemical Insecticide Residues and Household Surface Type on a *Beauveria bassiana-*Based Biopesticide (Aprehend^®^) for Bed Bug Management

**DOI:** 10.3390/insects12030214

**Published:** 2021-03-03

**Authors:** Ikkei Shikano, Giovani S. Bellicanta, Simona Principato, Nina E. Jenkins

**Affiliations:** 1 Department of Plant and Environmental Protection Sciences, University of Hawai‘i at Mānoa, Honolulu, HI 96822, USA; ishikano@hawaii.edu; 2 Department of Entomology, Pennsylvania State University, 107 Merkle Lab, University Park, PA 16802, USA; gsbellica@gmail.com (G.S.B.); simona.principato@gmail.com (S.P.); 3 Department of Crop and Environment Sciences, Harper Adams University, Newport, Shropshire TF10 8NB, UK; 4 Department of Entomology, University of Kentucky, S225 Ag Science–North, Lexington, KY 40546, USA

**Keywords:** biological control, *Cimex lectularius*, fungal entomopathogen, integrated pest management, pesticide, residual

## Abstract

**Simple Summary:**

Aprehend is a novel fungal biopesticide for the control of bed bug infestations. The formulation consists of fungal spores of the entomopathogen, *Beauveria bassiana* suspended in a proprietary oil formulation. Bed bug infestations are commonly treated with chemical insecticides and can require multiple applications to achieve eradication. The goal of this study was to determine if the presence of previously applied chemical insecticide residues would impact the efficacy of Aprehend when applied on top of chemical residues. A series of experiments was conducted by applying Aprehend on top of dried residues of 22 chemical insecticides on different surface types (fabric and wood). We found that residues of eight of the pesticides evaluated did not impact viability of the fungal spores in comparison to the control. Further evaluation of the effect of chemical residues on bed bug mortality revealed that chemical residues on fabric surfaces had less detrimental impact than those on wood. It is concluded that the practical implication of the presence of old chemical residues will have a minimal impact on overall control.

**Abstract:**

The biopesticide Aprehend, containing spores of the entomopathogenic fungus *Beauveria bassiana*, is a biological control agent for the management of the common bed bug (*Cimex lectularius* L.) (Hemiptera: Cimicidae). The spores are applied in strategically placed barriers, which bed bugs walk across as they search for a bloodmeal. Application of chemical insecticides by the general public and professional pest managers is common, which means that Aprehend may be sprayed on existing insecticide residues. We evaluated the effect of chemical residues, of 22 different chemical insecticides on different household surface types. We found that residues from 12 chemical pesticides significantly reduced spore viability measured 5 weeks after application in comparison to the control. However, efficacy of Aprehend, as measured by bed bug mortality and mean survival time after exposure to sprayed surfaces, seven weeks after application was not impacted detrimentally. Furthermore, in some cases, efficacy of old chemical residues was enhanced by the combination of chemical and Aprehend seven weeks after application. Surface type also played a role in the relative efficacy of all products and combinations, particularly as the residues aged.

## 1. Introduction

Management of infestations of the common bed bug, *Cimex lectularius* L. (Hemiptera: Cimicidae), most often involves the use of chemical insecticides [1,2,3]. This is despite the implementation of integrated pest management (IPM) approaches, which includes combined uses of numerous non-chemical methods, such as monitoring (e.g., visual inspections, traps, and canine scent detection), removing of household clutter, vacuuming, laundering, steaming, volumetric heating of rooms, spot freezing and mattress covers [4,5]. The heavy reliance on chemical insecticides likely stems from the cost of implementing non-chemical treatment alternatives, particularly heat, and the increased time required for training and implementation of non-chemical alternatives [4]. The goal of bed bug IPM is complete eradication of bed bugs from the infested area (i.e., home, office, apartment building, etc.). This is unlike IPM in other industries where the goal is to manage pests below levels that cause economic damage. Biopesticide products are of growing interest [4]. Botanical and non-chemical control methods such as the use of natural compound mixtures [6,7,8], microbial control using *Serratia marcescens*, *Pseudomonas fluorescens*, and *Bacillus thuringiensis israelensis* [9] and the use of entomopathogenic fungi like *Metarhizium anisopliae* [10,11] and *Beauveria bassiana* [12,13,14,15] are being investigated.

In 2017, a new biopesticide containing spores of the entomopathogenic fungus, *B. bassiana* (GHA) was registered by the US Environmental Protection Agency. Aprehend^®^ (ConidioTec, Centre Hall, PA, USA) is sprayed strategically to produce barriers (approximately 5 cm wide) of *B. bassiana* spores around bed frames, box springs (not mattresses) and items of furniture. These are areas that bed bugs are likely to walk when searching for a blood meal. Bed bugs pick up the spores on their tarsi and other body parts when walking on treated surfaces. Attachment to the bed bug’s body triggers the spores to germinate and penetrate the cuticle, eventually colonizing the body and causing death in 4 to 10 days [14,15]. After application, the *B. bassiana* spores persist and remain viable for up to 3 months, providing long-term protection from bed bug introductions (USA EPA, Pesticide Product Label, Aprehend, 27.03.2017).

However, the toxicity of chemical insecticides have been found to reduce the persistence and effectiveness of microbial insecticides (e.g., [16,17,18]). This is a significant concern for the successful integration of Aprehend into a professional bed bug management program since chemical insecticide residues are most likely to be present in the environment. In fact, most insecticide-based professional bed bug management programs require multiple visits [2], resulting in an accumulation of insecticide residues. Equally, if not more concerning is that the do-it-yourself (DIY) application of chemical insecticides in households is more common than treatments performed by professionals. Most residents with bed bugs in their homes attempt to control the bed bugs themselves before calling a professional [19]. Thus, there is a high probability that any bed bug infested environment where Aprehend might be applied will have residues of chemical insecticides. 

Moreover, the bed bug’s environment consists of a variety of surface types, including but not limited to bedding (fabric) materials, painted walls and baseboards, wooden furniture and carpet. Chemical insecticide residues on different household surface types have variable toxicity against insects, including bed bugs [20,21,22]. The efficacy of entomopathogenic fungal spores applied to different surfaces can also differ [14,23]. Bed bugs exposed to *B. bassiana* spores applied to jersey fabric died significantly faster than those exposed to spore-treated paper [14].

Here, we evaluated the effects of 16 professional and 6 DIY insecticidal sprays (Table 1) on the short and long-term viability of *B. bassiana* spores (Aprehend) and their effects on bed bug survival. The effects of all 22 insecticides on *B. bassiana* spore germination were screened on primed wooden boards. A subset of five professional-use insecticides, which are often used in combination with Aprehend by professional pest controllers, were further tested on box spring fabric and wooden furniture to assess their effects on spore germination and bed bug mortality. Another subset of one professional chemical and two DIY insecticides, which exhibited a strong detrimental effect on spore germination in the primed wooden board screening test, were further tested on box spring fabric to assess their effects on Aprehend efficacy against bed bugs and/or spore germination. It is important to note that the bed bugs were exposed to the Aprehend and/or insecticide-treated surfaces for 15 min. This exposure time was used for efficacy evaluation of Aprehend as part of the regulatory dossier submitted to the US Environmental Protection Agency (EPA). Lastly, we tested the potential detrimental effects of a commonly used adjuvant/synergist of chemical insecticides, piperonyl butoxide, on spore germination and Aprehend efficacy.

## 2. Materials and Methods

### 2.1. Bed Bugs

The bed bug colony was originally obtained from the University of Minnesota. It consists of a mixture of several field populations collected from cities across the U.S. in 2005, this colony has no documented resistance to chemical insecticides [14]. The bed bugs were maintained at high densities (>1000 individuals), consisting of multiple generations, in glass rearing jars containing folded poster boards for shelter, at 21 °C, 50% relative humidity (RH), and 14:10 (L:D) in a Percival (Perry, Iowa) environmental chamber. An artificial feeding system was used to offer weekly human blood meals. The adult bed bugs were fed two to four days prior to use in the experiments. 

### 2.2. Experiment 1 

**Figure 1:**
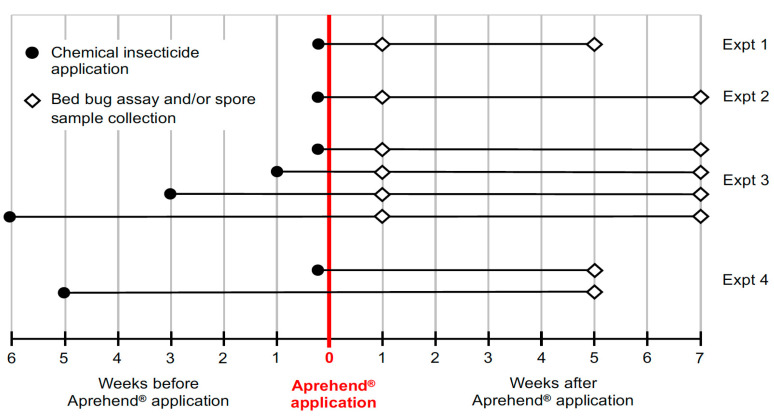
Experimental design summary showing time of application for chemical insecticides and Aprehend and duration of post-application monitoring for each experiment.

This experiment was conducted to determine the effect of dried residues of a wide range of chemical insecticides on the viability of *B. bassiana* spores. All 22 insecticides were applied to pre-primed wood (pine) boards purchased from Home Depot (Home Depot Product Authority, LLC. Atlanta, GA, USA). The insecticides were applied to the boards (12.5 × 30 cm) and permitted to air dry one day prior to the application of Aprehend. Spore samples were collected one and five weeks after Aprehend application to assess immediate and long-term effects of the chemicals on spore viability (Figure 1). Since all 22 insecticides could not be tested at once, the insecticides were tested in groups of four to seven. A board that was not treated with insecticide served as a control in each group. The inhibition of spore germination on each insecticide-treated board was estimated as the percent difference in spore germination relative to the spore germination on the control board.

### 2.3. Experiment 2 

Five chemical insecticides (PT Alpine, Bedlam Plus, CrossFire, Spectre 2 SC, and Temprid SC) commonly used in bed bug management programs by professional pest controllers were evaluated for their effect on spore viability and efficacy of Aprehend. The chemicals were applied to pieces of box spring fabric (17.5 × 30 cm; double-faced quilted cotton fabric; Jo-Ann Stores LLC, Hudson, OH, USA) and sections cut from a wooden bed frame (12.5 × 30 cm; from N.E.J.’s home; hereon referred to as “wooden furniture”) to test their effects on spore germination and bed bug mortality. The insecticides were applied and allowed to air dry, one day prior to the application of Aprehend to test the residual effects of the chemical residues. Spore germination and bed bug mortality were tested at one- and seven-weeks post Aprehend application (Figure 1). Fabric and furniture pieces that were not treated with insecticide or Aprehend and pieces that were only treated with Aprehend, served as negative and positive controls, respectively.

### 2.4. Experiment 3

Two insecticides (Raid and EcoRaider) that strongly inhibited spore germination in Experiment 1 were applied to box spring fabric (17.5 × 30 cm) and tested for their effects on spore germination and bed bug mortality. The insecticides were applied one day, 1 week, 3 weeks, and 6 weeks prior to the application of Aprehend to test how long the chemical residues remain active against Aprehend. Samples of Aprehend spores were collected one and seven weeks after Aprehend application (Figure 1). A fabric piece that was not treated with insecticide or Aprehend and a fabric piece that was only treated with Aprehend, served as negative and positive controls, respectively.

### 2.5. Experiment 4

This experiment aimed to evaluate impact of Zenprox Xtend Aerosol, and the synergist piperonyl butoxide which is a component of Zenprox. This product had significantly reduced spore germination in Experiment 1. Zenprox was applied to box spring fabric (17.5 × 30 cm), one day and 5 weeks before Aprehend and percent spore germination was assessed 5 weeks after Aprehend application (Figure 1). A fabric piece that was not treated with Zenprox served as a positive control for spore germination. Piperonyl butoxide was sprayed on box spring fabric at concentrations of 1.5% (concentration in Zenprox) and 5% diluted in 50% ethanol one day prior to Aprehend application. Effects on spore germination and bed bug mortality were assessed 5 weeks after Aprehend application. A fabric piece that was treated with only 50% ethanol served as the negative control, and a fabric piece that was treated with 50% ethanol and Aprehend served as the positive control. 

### 2.6. Application of Insecticides

All chemical insecticides were applied to cover the entire surface of the treated material, following instructions on the product labels. For products that provided high and low application rates, we used the high application rate except for Temprid SC. All materials (primed wood, wooden furniture, and fabric pieces) were weighed before and after insecticide application. Concentrated insecticide formulations that needed to be diluted to the appropriate label rate were sprayed on surfaces using the aerosol-based spray system, Preval Sprayer (Nakoma Products, LLC., Bridgeview, IL, USA).

### 2.7. Application of Aprehend

The surface to be treated was hung (fabric) or placed (wood and furniture) at the back of a reverse flow Hepa filter cabinet. Aprehend was applied using a low-volume low-pressure spray applicator (ConidioTec LLC, Centre Hall, PA, USA) held approximately 4” (20 cm) from the surface, moving the gun horizontally across the surface at a speed of one linear foot per second (30.5 cm s^−1^) as described on the product label. The Aprehend-treated material and untreated (unsprayed) material were stored on racks under a laboratory bench, which was curtained off with a black plastic sheet to create a realistic low-light environment. The laboratory was temperature-controlled at 21 °C. All materials (wood, furniture, and fabric pieces) were weighed before and after Aprehend application to confirm the volume application rate. 

### 2.8. Survival of Spores

Samples of Aprehend spores were collected from three different arbitrarily selected spots (2 × 2 cm) on the treated primed wood, wooden furniture and fabric pieces to account for any potential uneven application of insecticide and Aprehend. For box spring fabric, three 2 × 1 cm pieces were cut and removed from the swatch. For wood and furniture, three 2 × 1 cm areas were swabbed with individual sterile cotton tipped applicators (Puritan; Puritan Medical Products Company LLC., Guilford, ME, USA) that were moistened with Isopar M (ExxonMobil Chemical Company, Houston, TX, USA). The fabric sample and swab tip sample were placed in individual glass vials with 1 ml of Isopar M. The vials were vortexed for 1 min, followed by sonication for 1 min to release the spores from the fabric and swab. A 10 μL droplet of the resulting spore suspension from each sample was pipetted onto a Sabouraud dextrose agar plate. The plate was tilted in a circular motion to gently spread each droplet. The plate was placed in an incubator at 26.5 °C for 20 h. For each droplet, the numbers of germinated and non-germinated spores were counted at ×400 magnification using a phase contrast microscope until a minimum of 300 spores was counted.

### 2.9. Bed bug Survival Assay

The effects of insecticide residues and Aprehend on bed bug survival were evaluated in Experiment 2, 3, and 4 on the same day(s) as spore sample collection. In Experiment 3, a bed bug survival assay was only conducted at one-week post Aprehend application. In Experiment 4, a bed bug survival assay was only conducted for the piperonyl butoxide treatments. Thirty recently fed (2–4 d before assay), mixed-sex, adult bed bugs were placed in groups of 10 on an arbitrarily selected area of the insecticide and/or Aprehend-treated material or control material. Each group of bed bugs were contained by placing the lid of a 10 cm diameter glass petri dish on top. The bed bugs were allowed to move freely under the lid for 15 min. They were then transferred to a 1 oz plastic portion cup (with lid). A folded filter paper shelter was provided in each cup. Cups were maintained on the laboratory bench and mortality of bed bugs was monitored daily for 14 d. Daily monitoring was conducted by tapping each pot on the bench to dislodge dead bed bugs from the paper shelter. Dead bed bugs were then removed and verified as dead by prodding. The number of cadavers recovered was recorded for each day. 

### 2.10. Statistical Analysis 

The germination of *B. bassiana* spores were analyzed by generalized linear model using a binomial distribution. The survival times of bed bugs were compared by Kaplan–Meier survival estimator. Mean and median survival times were also obtained from the Kaplan–Meier estimator. Bed bugs that survived beyond the 14-day observation period were coded as right censored. As estimation of mean survival time becomes biased when more than 30% of the data is censored [24], mean survival time was only estimated for treatments that produced more than 70% mortality. The Kaplan–Meier estimator of the median survival time is minimally biased by censoring [24]. All analyses were performed on JMP Pro 14 (SAS Institute, Cary, NC, USA).

## 3. Results

### 3.1. Experiment 1

Of the 22 insecticide residues tested, residues of five insecticides significantly reduced spore germination at one-week post Aprehend application and 12 insecticides at five weeks post application (Table 1). Residues of all six of the DIY insecticides, including two “natural” plant essential oil-based insecticides, significantly reduced spore germination at five weeks post Aprehend application. Residues of six professional insecticides (out of 16) reduced spore germination. These included Suspend and D-Force, both of which contain deltamethrin, Zenprox and Precor, which contain the same combination of active ingredients (Table 1), Gentrol, which contains the insect growth-regulator (S)-Hydroprene, and Optimate, which contains the pyrethroid compound γ-cyhalothrin. Residues of other products that contain cyhalothrin (Black Flag, Demand and Hot Shot) also significantly or marginally reduced spore germination at five weeks post Aprehend application. Residues of products containing some other pyrethroid compounds were also associated with significantly reduced spore germination at five weeks post Aprehend application, including cyfluthrin, deltamethrin, imiprothrin, tetramethrin, and pyrethrins. However, residues of products containing dinotefuran, prallethrin, esfenvalerate, imidacloprid, chlorfenapyr, dichlorvos, chlothianidin, cold-pressed neem oil, phenothrin, metofluthrin, and bifenthrin did not significantly reduce spore germination (Table 1).

### 3.2. Experiment 2

Box spring fabric: Residues of Alpine, Bedlam Plus, CrossFire, Spectre, and Temprid one week (+1 day) after application on fabric were all highly effective against bed bugs (80–100% mortality; Table 2) after 15 min of exposure. Aprehend spores applied to any of the five insecticide residues germinated at comparable levels to spores on untreated fabric one week (83–88%) and seven weeks (64–69%) after Aprehend application (Table 2). All bed bugs died when exposed to fabric co-treated with Aprehend and any of the five insecticides. Bed bugs died significantly faster when exposed to Spectre and Temprid residues that had been co-treated with Aprehend compared to either insecticide residue alone. 

Seven-week (+1 day) old residues of CrossFire and Temprid on fabric were still effective against bed bugs (97–100% mortality). Residues of Alpine and Spectre were only moderately effective (57% mortality) and residues of Bedlam Plus were ineffective against bed bugs that were exposed to the residues for just 15 min. However, 100% of bed bugs died on seven-week old insecticide residues of all five insecticides if they had been co-treated with Aprehend. Seven-week old Aprehend alone (i.e., positive control) also killed 97% of bed bugs.

Wooden furniture: Residues of Temprid one week (+1 day) after application on wooden furniture killed 100% of bed bugs. However, residues of the other four insecticides only killed 10–30% of bed bugs after a 15 min exposure period (Table 3). Percent germination of spores after one week on the five insecticide residues were comparable (78–82%) to spores on the control (Table 3). Co-treatment of the insecticide residues with Aprehend significantly increased overall bed bug mortality (except on Temprid residue where mortality was 100% with and without Aprehend). 

Only 29% of seven-week old Aprehend spores on residue-free wooden furniture (positive control) germinated. However, this was still enough to kill 76% of bed bugs that were exposed to the positive control. Percent germination of spores after seven weeks on any of the insecticide residues were significantly lower than the positive control (11–17%). This lower germination was associated with significantly lower bed bug mortality on Aprehend-treated residues of Alpine, Bedlam Plus, and Spectre, compared to Aprehend on residue-free furniture. However, application of Aprehend to residues of Alpine and Spectre still produced significantly higher bed bug mortality than the insecticide residues alone, indicating that there were enough viable spores to induce mortality.

### 3.3. Experiment 3

EcoRaider: A 15 min exposure to residues of EcoRaider alone on box spring fabric did not reduce bed bug survival, except for the most recently treated residue (8-d old residue; Table 4). However, EcoRaider residues of up to six weeks old, significantly reduced the germination of subsequently applied Aprehend spores. The percent mortality of bed bugs induced by the combination of Aprehend on EcoRaider residues was unaffected at one week after Aprehend application (100% mortality) and only slightly reduced at seven weeks after Aprehend application (87–97% mortality), compared to Aprehend alone (100% mortality at one and seven weeks). However, the speed of kill was significantly slower when bed bugs were exposed to Aprehend on EcoRaider residues.

Raid: The effectiveness of Raid residues on box spring fabric in killing bed bugs decreased with increasing residue age; residues up to two weeks old resulted in 93-100% mortality (Table 5). The Raid residues significantly reduced the viability of Aprehend spores. However, in contrast to the detrimental effects of EcoRaider residues on Aprehend-induced bed bug mortality, Aprehend applied to Raid residues killed bed bugs faster than Aprehend (Table 5, column: “Mean survival time”, *p* < 0.001 for all Raid residue ages with Aprehend relative to Aprehend on control fabric) or Raid alone (Table 5, column: “Effect of adding Aprehend”, *p* < 0.001 for all Raid residue ages). Given that Aprehend kills more slowly than Raid and the combination of the two insecticides killed bed bugs more quickly than Raid alone, this suggests that Aprehend had an enhancing effect on the efficacy of the Raid residues. Percent germination of spores exposed to Raid residues for seven weeks was 58, 69, 67, and 68% on one-day, one-week, three-week, and six-week old Raid residues, respectively, while germination on the positive control was 75% (Table 6). A bed bug survival assay was not conducted at the seven-week time point.

### 3.4. Experiment 4

Compared to the germination of positive control spores on box spring fabric (87%), germination was significantly reduced when applied to one-day and five-week old Zenprox residues (73 and 80% germination, respectively; *p* < 0.0001). Residues of piperonyl butoxide at 5 and 1.5% on box spring fabric also significantly reduced spore germination (Table 7). Bed bug survival was marginally longer when bed bugs were exposed to Aprehend on piperonyl butoxide than Aprehend alone.

## 4. Discussion

The initial screen of 22 insecticides revealed that residues of more than half of the insecticides were capable of reducing the germination of Aprehend spores, when spores were exposed to the chemical residues for five weeks. Residues of all six of the DIY products reduced spore germination. Previous authors have reported detrimental effects of chemical pesticides on entomopathogenic fungi [16,17,18]. However, most studies that evaluate the effect of chemicals on fungal viability focus on measuring the immediate toxic effect of chemicals on conidial germination [25]. Measuring the effect of exposure to chemical residues on conidial viability over time is important for products that require long-term residual efficacy such as Aprehend. Even when Aprehend was applied to six-week old residues of Raid and EcoRaider and five-week old residues of Zenprox (professional-use), spore viability was significantly reduced. In fact, even residues of just the insecticide synergist piperonyl butoxide found in many insecticide products, such as Zenprox, significantly reduced spore viability. Chemical insecticide applications are often applied by professionals, and residents frequently attempt to control bed bug infestations themselves using DIY products before calling a professional [19]. Thus, chemical insecticide residues are highly likely to be present before Aprehend is used. 

However, the detrimental effects of insecticide residues on fungal spore survival did not necessarily result in reduced mortality of bed bugs exposed to Aprehend on the insecticide-treated surfaces. Fortunately, we found that the only residue that reduced spore germination and prolonged survival of bed bugs was the DIY-insecticide, EcoRaider. In contrast, when Aprehend was applied to residues of Raid, which reduced spore germination, efficacy was increased. When Aprehend was applied to Raid residues, regardless of the age of the residue, mortality of bed bugs was higher and/or occurred faster than with either product alone. Synergistic effects of combining pyrethroids and mycoinsecticides has been found previously [26,27,28]. It is possible that the synergism could arise from one insecticide, chemical or fungal, weakening the insect’s resistance to the other insecticide, especially if exposure to each insecticide was not simultaneous [28]. However, in our study, the bed bugs contacted Aprehend and Raid simultaneously and median time to death was a little over 24 h, which is only enough time for the Aprehend spores to start germinating. Thus, it is unlikely that fungal infection could have played a role in the enhanced mortality on Raid residues. A more plausible explanation is that the carrier solution in Aprehend, which contains petroleum distillates, may have activated or enhanced the Raid residue. Other studies found synergy between pyrethroid and fungal insecticides if the fungal product contained petroleum distillates as a carrier [26,27]. Wu et al. [27] suggested that the petroleum distillates may increase the speed of chemical penetration through the cuticle and the proportion of toxicant entering the body. The addition of mineral oil with permethrin was previously shown to increase the rate of permethrin penetration in the carrot weevil, *Listronotus oregonensis* [29]. Studies on pyrethroid resistant mosquitoes have shown a synergistic effect of plant essential oils in combination with pyrethroid insecticides [30,31]. However, these authors did not use simple oils or petroleum distillates as part of their investigations. Bateman et al. [32], originally described enhanced infectivity of entomopathogenic fungi formulated in oil in comparison to water based formulations. Dang et al. [22] also described the enhanced activity of chemical insecticides when formulated in various oils in comparison to acetone. Our data corroborate this finding. 

The type of surface material treated with five commonly used professional chemical insecticides followed by Aprehend influenced the compatibility of the products. First, in the absence of chemical insecticide residues, the viability of Aprehend spores declined more rapidly on wooden furniture than on box spring fabric during the same period of time. However, a decline in spore viability did not translate to reduced bed bug mortality on either surface type, suggesting that enough viable spores remained on both surface types after seven weeks to kill exposed bed bugs. Generally, residues of the chemical insecticides were less effective in killing bed bugs (after a 15 min exposure) on treated wooden furniture than on box spring fabric. The importance of surface type on the efficacy of residual chemical insecticides has been demonstrated by multiple authors [20,21,22]. Dang et al. [22], demonstrated that efficacy of chemical insecticides was best on smooth surfaces, such as glass in comparison to rough, or absorbent surfaces such as filter paper. These authors proposed that this difference was likely due to a combination of crystallization of the chemical actives on absorbent surfaces and a reduction in availability of the insecticide due to absorption. Conversely, bed bug mortality was faster following exposure to one-week old residues of Aprehend on box spring fabric (MST 4.57 days) in comparison to wood (MST 7.11 days). These results confirm those already reported [14,23]. Unlike chemical actives, *B. bassiana* is particulate, and does not crystalize, and the spores themselves are less likely to be absorbed into the fabric. 

Efficacy of all five chemical residues declined over time on wood and box spring fabric. On the wooden furniture, the addition of Aprehend to the insecticide residues generally increased bed bug mortality in comparison to the chemical insecticides alone. Furthermore, the addition of Aprehend enhanced the efficacy of chemical residues, both in terms of total mortality and speed of kill. The mean survival time for chemicals on box spring fabric in combination with Aprehend was faster than for Aprehend alone. This again suggests that the oil formulation may be responsible for enhancing efficacy of the chemical products, rather than the fungal pathogen itself. 

As discussed, surface type has been shown to be an important factor in the efficacy of fungal insecticides, but also their persistence [33]. Here we observed that surface type can also influence the interactive effects of chemical and fungal insecticides. Residues of Zenprox Xtend reduced spore viability by 61% on primed boards, but only by 16% on box spring fabric five weeks after application. This difference in interaction could be due to the release of toxins from the primer used on the wooden boards, whether caused by the active ingredient or the formulating ingredients. 

Since it is often impossible for pest management professionals to know which products (if any) have been previously applied in any given situation, these results will provide a greater degree of confidence. It is unlikely that the presence of any of the products evaluated here will result in complete inactivation of Aprehend, and in many cases, existing residues may result in faster kill of bed bugs exposed to the combination of old chemical residues and Aprehend. The only exception to this may be prior use of EcoRaider, which did impact both viability and efficacy of Aprehend even when the residues were three weeks old. This is unfortunate, since recent research into essential oils for bed bug control has demonstrated their potential utility as alternatives to conventional chemicals for bed bug control and resistance management [6,8]. Unlike their observed synergistic effect with chemical insecticides [30], essential oils appear to have a detrimental impact on *B. bassiana* spores. However, providing these products are utilized in separate treatment areas, we would not anticipate any detrimental effect. 

Finally, this study was limited to investigating the effect of single product formulations on Aprehend. Further work will be required to determine the potential impact of combinations of multiple chemical residues on the efficacy of Aprehend. However, our results to date, in addition to previously published work on the effect of pyrethroid coated mattress liners [34,35], should provide pest management professionals with reasonable confidence in the use of Aprehend even in situations where existing chemical residues are likely to be present. 

## Figures and Tables

**Table 1:** List of professional and do-it-yourself (DIY) insecticide products, their active ingredients, and the effects of their residues on the percent germination of *Beauveria bassiana* spores, one and five weeks after Aprehend^®^ application. Values represent the percent difference in spore germination relative to germination on primed boards that were not previously treated with an insecticide. Negative values represent inhibition of spore germination.

Percent Difference in Spore Germination Relative to Untreated Control				
			Time Post Aprehend Application	
Professional Products	Formulation	Active Ingredients ^a^	1 Week	5 Weeks
PT^®^ Alpine^®^ Pressurized Insecticide	Aerosol	Dinotefuran, 0.25% Pyriproxyfen, 0.1% Prallethrin, 0.05%	–2	–2
Bedlam Plus^®^	Aerosol	Imidacloprid, 0.05% MGK 264, 1% Phenothrin, 0.4%	–5	–2
Cirkil^®^ RTU	Ready-to-use spray	Cold-pressed neem oil, 5.5%	+7	–2
Cross Fire^®^ Bed Bug Concentrate	Concentrate	Clothianidin 0.4% Metofluthrin 0.01% Piperonyl butoxide 1%	+1	+3
D-Force^®^	Aerosol	Deltamethrin, 0.06%	+4	–26 *
Demand^®^ CS	Capsule suspension	λ-Cyhalothrin, 0.03%	–15	–19^†^
Fenvastar Plus^TM^	Concentrate	Esfenvalerate, 0.05%	+1	–8
Gentrol^®^ IGR Concentrate	Concentrate	(S)-Hydroprene, 0.07%	–2	–25 **
Nuvan^®^ Directed Spray^TM^ Aerosol	Aerosol	Dichlorvos, 0.5%	–7 ^†^	–3
Optimate^®^ CS Controlled Release Insecticide	Capsule suspension	γ-Cyhalothrin, 0.015%	0	–25 *
Precor^®^ 2625 Premise Spray	Aerosol	Etofenprox, 1% Tetramethrin, 0.25% Pyrethrins, 0.15% Piperonyl Butoxide, 1.50% (S)-Methoprene, 0.09%	–5	–31 *
Spectre^®^ 2 SC	Suspension concentrate	Chlorfenapyr, 0.5%	–2	–7
Suspend^®^ SC Insecticide	Suspension concentrate	Deltamethrin, 0.06%	–36 **	–39 *
Temprid^®^ SC	Suspension concentrate	Imidacloprid, 0.05% β-Cyfluthrin, 0.025%	+2	+5
Transport^®^ Mikron^TM^ Insecticide	Concentrate	Acetamiprid, 0.05% Bifenthrin, 0.06%	–4 **	–14 ^†^
Zenprox^®^ Xtend Aerosol	Aerosol	Etofenprox, 1% Tetramethrin, 0.25% Pyrethrins, 0.15% Piperonyl Butoxide, 1.50% (S)-Methoprene, 0.09%	–7	–61 *
**DIY Products**				
Bayer Advanced^®^ Home Pest Bed Bug & Flea Killer	Aerosol	Imidacloprid, 0.025% β-Cyfluthrin, 0.0125%	–5 ^†^	–26 **
Black Flag^®^ Flea & Tick Aerosol	Aerosol	γ-Cyhalothrin, 0.005% Pyriproxyfen, 0.016%	–13 *	–32 *
EcoRaider^®^ Natural Bed Bug Killer	Ready-to-use spray	Natural geraniol, 1% Natural cedar oil, 1% Sodium lauryl sulfate, 2%	–2	–23 *
EcoVia^TM^ CA	Aerosol	Thyme oil, 0.88% Rosemary oil, 0.53% Cinnamon oil, 0.26%	–16 **	–27 *
Hot Shot^®^ Bed Bug Killer	Aerosol	Imiprothrin, 0.1% λ-Cyhalothrin, 0.025%	–7	–35 **
Raid^®^ Ant & Roach	Aerosol	Imiprothrin, 0.06% Cypermethrin, 0.1%	–16 **	–43 ***

^a^ Active ingredient concentrations at label rates; ^†^ *p* < 0.10; * *p* < 0.05; ** *p* < 0.01; *** *p* < 0.001.

**Table 2:** Bed bug mortality following 15 min exposure, and Aprehend^®^ spore viability on residues of five professional-use insecticides on box spring fabric (Experiment 2).

Time Post Aprehend^®^ Application	Insecticide ^a^	No Aprehend^®^			With Aprehend^®^				
		Mortality	Mean Survival Time (± SE) ^bc^	Median Survival Time	Mortality	Mean Survival Time (± SE) ^bc^	Median Survival Time	Effect of Adding Aprehend^® d^	Spore Viability ^e^
1 week	PT^®^ Alpine^®^	94%	3.16 ± 0.57 ***	2	100%	1.87 ± 0.18 ***	2	*X^2^* = 3.23	84%
	Bedlam Plus^®^	80%	3.60 ± 0.68 ***	2	100%	2.57 ± 0.24 ***	2	*X^2^* = 0.87	88%
	CrossFire^®^	100%	2.39 ± 0.23 ***	2	100%	2.17 ± 0.20 ***	2	*X^2^* = 0.79	87%
	Spectre^®^ 2 SC	83%	5.60 ± 0.82 ***	3	100%	2.30 ± 0.24 ***	2	*X^2^* = 11.92 ***	87%
	Temprid^®^ SC	100%	1.13 ± 0.06 ***	1	100%	1.00 ± 0.00 ***	1	*X^2^* = 4.21 *	83%
	None	10%	-	>14	100%	4.57 ± 0.18	5	*X^2^* = 63.80 ***	84%
7 weeks	PT^®^ Alpine^®^	57%	-	8 ***	100%	3.33 ± 0.29 ***	3	*X^2^* = 27.94 ***	69%
	Bedlam Plus^®^	10%	-	>14	100%	3.53 ± 0.39 ***	4	*X^2^* = 60.54 ***	64%
	CrossFire^®^	97%	4.03 ± 0.60	3 ***	100%	3.37 ± 0.22 ***	3	*X^2^* = 0.25	65%
	Spectre^®^ 2 SC	57%	-	12 ***	100%	4.70 ± 0.43	5	*X^2^* = 23.76 ***	69%
	Temprid^®^ SC	100%	2.06 ± 0.40	1 ***	100%	1.70 ± 0.20 ***	1	*X^2^* = 0.58	69%
	None	7%	-	>14	97%	5.97 ± 0.35	5	*X^2^* = 59.32 ***	67%

^a^ Insecticides were applied one day prior to Aprehend^®^ application. ^b^ Comparison of Kaplan–Meier survival curves for each insecticide relative to the “None” control (with or without Aprehend^®^, respectively), using log-rank chi-square test (* *p* < 0.05, *** *p* < 0.001). Number of bed bugs tested per treatment = 29–31. ^c^ A dash denotes that estimation could not be performed because of low mortality. ^d^ Comparison of Kaplan–Meier survival curves between Aprehend^®^-treated and not treated for each insecticide using log-rank chi-square test (* *p* < 0.05, *** *p* < 0.001). ^e^ Percent spore germination was not significantly lower than the no insecticide residue control (*p* > 0.05).

**Table 3:** Bed bug mortality following 15 min exposure, and Aprehend^®^ spore viability on residues of five professional-use insecticides on wooden furniture (Experiment 2).

Time Post Aprehend^®^ Application	Insecticide ^a^	No Aprehend^®^			With Aprehend^®^				
		Mortality	Mean Survival Time (± SE) ^bc^	Median Survival Time	Mortality	Mean Survival Time (± SE) ^bc^	Median Survival Time	Effect of Adding Aprehend^® d^	Spore Viability ^e^
1 week	PT^®^ Alpine^®^	33%	- *	>14	80%	8.20 ± 0.59	6.5	*X*^2^ = 12.80 ***	82%
	Bedlam Plus^®^	23%	-	>14	71%	9.32 ± 0.60 *	8	*X*^2^ = 16.15 ***	78%
	CrossFire^®^	33%	-*	>14	83%	7.55 ± 0.72	6	*X*^2^ = 17.30 ***	79%
	Spectre^®^ 2 SC	10%	-	>14	97%	7.50 ± 0.59	6.5	*X*^2^ = 51.27 ***	80%
	Temprid^®^ SC	100%	2.13 ± 0.36 ***	1	100%	1.97 ± 0.33 ***	1	*X*^2^ = 0.30	81%
	None	10%	-	>14	83%	7.11 ± 0.31	6	*X*^2^ = 34.16 ***	82%
7 weeks	PT^®^ Alpine^®^	13%	-	>14	53%	-**	13	*X*^2^ = 12.24 ***	17% ***
	Bedlam Plus^®^	23%	-	>14	33%	-***	>14	*X*^2^ = 0.72	12% ***
	CrossFire^®^	57%	-***	11	67%	-	10	*X*^2^ = 0.26	12% ***
	Spectre^®^ 2 SC	20%	-	>14	50%	-**	≥14	*X*^2^ = 5.35 *	11% ***
	Temprid^®^ SC	87%	6.37 ± 0.82 ***	6	90%	5.67 ± 0.67 *	5	*X*^2^ = 0.25	14% ***
	None	7%	-	>14	76%	7.28 ± 0.43	7	*X*^2^ = 30.23 ***	29%

^a^ Insecticides were applied one day prior to Aprehend application. ^b^ Comparison of Kaplan–Meier survival curves for each insecticide relative to the “None” control (with or without Aprehend^®^, respectively), using log-rank chi-square test (* *p* < 0.05, ** *p* < 0.01, *** *p* < 0.001). Number of bed bugs tested per treatment = 29–31. ^c^ A dash denotes that estimation could not be performed because of low mortality. ^d^ Comparison of Kaplan–Meier survival curves between Aprehend^®^-treated and not treated for each insecticide using log-rank chi-square test (* *p* < 0.05, ** *p* < 0.01, *** *p* < 0.001). ^e^ Percent spore germination significantly lower compared to the no insecticide residue control (^*^p < 0.001).

**Table 4:** Bed bug mortality following 15 min exposure, and Aprehend^®^ spore viability on residues of EcoRaider^®^ on box spring fabric (Experiment 3).

Time Post Aprehend^®^ Application	Age of EcoRaider^®^ Residue at Aprehend^®^ Application ^a^	No Aprehend^®^			With Aprehend^®^			
		Mortality	Mean Survival Time (± SE) ^bc^	Median Survival Time	Mortality	Mean Survival Time (± SE) ^dc^	Median Survival Time	Spore Viability ^e^
1 week	1 day	43%	-*	>14	100%	5.90 ± 0.30 ***	6	64% ***
	1 week	20%	-	>14	100%	5.97 ± 0.26 ***	5.5	75% ***
	3 weeks	23%	-	>14	100%	5.80 ± 0.21 ***	5.5	75% ***
	6 weeks	23%	-	>14	100%	5.57 ± 0.37 **	5	75% ***
	None	13%	-	>14	100%	4.80 ± 0.13	5	86%
7 weeks	1 day	7%	-	>14	87%	8.00 ± 0.53 **	7	56% ***
	1 week	23%	-	>14	90%	7.83 ± 0.53 **	8	63% ***
	3 weeks	7%	-	>14	90%	8.23 ± 0.57 **	7	69% **
	6 weeks	17%	-	>14	97%	6.77 ± 0.41	6	72%
	None	7%	-	>14	100%	6.13 ± 0.45	6	75%

^a^ Insecticides were applied one day prior to Aprehend^®^ application. ^b^ Comparison of Kaplan–Meier survival curves for each EcoRaider^®^ residue age relative to the “None” control, using log-rank chi-square test (* *p* < 0.05, ** *p* < 0.01, *** *p* < 0.001). Number of bed bugs tested per treatment = 29–31. ^c^ A dash denotes that estimation could not be performed because of low mortality. ^d^ Comparison of Kaplan–Meier survival curves for each EcoRaider^®^ residue age treated with Aprehend^®^ relative to the positive control (Aprehend^®^ only), using log-rank chi-square test (* *p* < 0.05, ** *p* < 0.01, *** *p* < 0.001). Significant values indicate negative effect of EcoRaider^®^ residues on Aprehend^®^ efficacy. Number of bed bugs tested per treatment = 29–31. ^e^ Percent spore germination compared to the no insecticide residue control (* *p* < 0.05, ** *p* < 0.01, *** *p* < 0.001).

**Table 5:** Bed bug mortality following 15 min exposure, and Aprehend^®^ spore viability on residues of Raid^®^ on box spring fabric (Experiment 3). Bed bug mortality was not assessed on the 7-week old Aprehend-treated fabric due to unavailability of bed bugs.

Time post Aprehend^®^ Application	Age of Raid^®^ Residue at Aprehend Application ^a^	No Aprehend^®^			With Aprehend^®^				
		Mortality	Mean Survival Time (± SE) ^ab^	Median Survival Time	Mortality	Mean Survival Time (± SE) ^ab^	Median Survival Time	Effect of Adding Aprehend^® c^	Spore Viability ^d^
1 week	1 day	100%	2.37 ± 0.39 ***	2	100%	1.17 ± 0.08 ***	1	*X*^2^ = 13.26 ***	71% ***
	1 week	93%	3.33 ± 0.45 ***	2	100%	1.27 ± 0.08 ***	1	*X*^2^ = 21.64 ***	73% ***
	3 weeks	43%	-**	>14	100%	2.57 ± 0.28 ***	2	*X*^2^ = 45.13 ***	73% ***
	6 weeks	30%	-	>14	100%	3.13 ± 0.33 ***	2.5	*X*^2^ = 58.26 ***	81% **
	None	13%	-	>14	100%	4.80 ± 0.13	5	*X*^2^ = 57.42 ***	86%
7 week	1 day								58% ***
	1 week								69% **
	3 weeks								67% ***
	6 weeks								68% ***
	None								75%

^a^ Comparison of Kaplan–Meier survival curves for each residue age relative to the “Untreated” control, using log-rank chi-square test (** *p* < 0.01, *** *p* < 0.001). Therefore, encompasses survival percentage and time. Number of bed bugs tested per treatment = 30. ^b^ A dash denotes that estimation could not be performed because of low mortality. ^c^ Comparison of Kaplan–Meier survival curves between Aprehend^®^-treated and not treated for each Raid^®^ residue age, using log-rank chi-square test (** *p* < 0.01, *** *p* < 0.001). ^d^ Percent spore germination compared to the no insecticide residue control (** *p* < 0.01, *** *p* < 0.001).

**Table 6:** Aprehend^®^ spore viability on residues of Zenprox^®^ Xtend Aerosol on box spring fabric (Experiment 4).

Insecticide	Time Post Aprehend^®^ Application	Age of Insecticide Residue at Aprehend^®^ Application	Spore Viability ^a^
Zenprox^®^	5 weeks	1 day	73% ***
		5 weeks	80% ***
		None	87%

^a^ Percent spore germination compared to the no insecticide residue control (*** *p* < 0.001).

**Table 7:** Bed bug mortality and Aprehend^®^ spore viability on residues of piperonyl butoxide (PB) on box spring fabric (Experiment 4). Aprehend was applied one day after PB application, and bed bug mortality and spore viability were assessed after 5 weeks.

Treatment	Mortality ^a^	Mean Survival Time (± SE) ^bc^	Median Survival Time	Spore Viability ^d^
PB 5% + Aprehend^®^	100%	5.17 ± 0.14 ^†^	5	68% ***
PB 1.5% + Aprehend^®^	100%	5.23 ± 0.14 *	5	71% ***
Aprehend^®^	100%	4.73 ± 0.17	4.5	87%
Untreated	13%	-***	>14	n/a

^a^ Number of bed bugs tested per treatment = 30. ^b^ Comparison of Kaplan–Meier survival curves of the piperonyl butoxide treatments and untreated box spring fabric against the Aprehend^®^ only treatment, using log-rank chi-square test (^†^ *p* < 0.10, * *p* < 0.05, *** *p* < 0.001). ^c^ A dash denotes that estimation could not be performed because of low mortality. ^d^ Percent spore germination compared to the no PB residue control (* *p* < 0.05, *** *p* < 0.001).

## Data Availability

The data presented in this study are available on request from the corresponding author.
